# Complete plastome sequence of *Callicarpa nudiflora* Vahl (Verbenaceae): a medicinal plant

**DOI:** 10.1080/23802359.2019.1613197

**Published:** 2019-07-10

**Authors:** Hong-Xin Wang, Lin Chen, Xia-Lan Cheng, Wen-Shu Chen, Lin-Ming Li

**Affiliations:** a Institute Arts, Sanya University, Sanya, Hainan, China;; b School of Life Science and Technology, Lingnan Normal University, Zhanjiang, Guangdong, China;; c Hainan Provincial Forestry Project Management Office, Haikou, Hainan, China;; d College of Forestry, Hainan University, Haikou, Hainan, China

**Keywords:** *Callicarpa nudiflora* Vahl, plastome, phylogeny, genome structure, Verbenaceae

## Abstract

*Callicarpa nudiflora* Vahl is a medicinal plant occurring in Guangdong, Guangxi, Hainan provinces of China. Here, we report and characterize the complete plastid genome sequence of *C. nudiflora* in an effort to provide genomic resources useful for promoting its conservation. The complete plastome is 154,080 bp in length and contains the typical structure and gene content of angiosperm plastome, including two inverted repeat (IR) regions of 25,657 bp, a large single-copy (LSC) region of 84,949 bp, and a small single-copy (SSC) region of 17,817 bp. There are 113 genes annotated, including 79 unique protein-coding genes, 4 unique ribosomal RNA genes, and 30 transfer RNA genes. To investigate the evolution status of *C. nudiflora*, as well as Verbenaceae, we constructed a phylogenetic tree with *C. nudiflora* and other 11 species based on their complete chloroplast genomes. According to the phylogenetic topologies, *C. nudiflora* was closely related to *Lancea hirstua*.

*Callicarpa nudiflora* Vahl is a plant of the family Verbenaceae. It is widely distributed in Guangdong, Guangxi, Hainan provinces of China and it grows in the mixed forests with altitude from 800 to 1000 m (Chen and MIchael 1994). It has high medicinal value (Huang et al. 2014). So far, there have been no studies on the genome of *C. nudiflora.* Consequently, the genetic and genomic information is urgently needed to promote its systematics research and the development of conservation value of *C. nudiflora.* Here, we report and characterize the complete plastid genome sequence of *C. nudiflora* (GenBank accession number: MK783316) in an effort to provide genomic resources useful for promoting its conservation.

In this study, *C. nudiflora* was sampled from Sanlingshan of Zhanjiang city Nature Reserve in Guangdong province of China (110.40°E, 21.18°N). A voucher specimen (Cheng, CL3) was deposited in the Herbarium of Lingnan Normal University, Zhanjiang, China.

The experiment procedure is as reported in Zhu et al. (2018). Around 6 Gb clean data were assembled against the plastome of *Premna microphylla* (KM981744.1) (Zhang and Handy ) using MITO bim v1.8 (Natural History Museum, University of Oslo, Oslo, Norway) (Hahn et al. 2013). The plastome was annotated using Geneious R8.0.2 (Biomatters Ltd., Auckland, New Zealand) against the plastome of *P. microphylla* (KM981744.1). The annotation was corrected with DOGMA (Wyman et al. 2004).

The plastome of *C. nudiflora* was found to possess a total length 154,080 bp with the typical quadripartite structure of angiosperms, containing two inverted repeats (IRs) of 25,657 bp, a large single-copy (LSC) region of 84,949 bp, and a small single-copy (SSC) region of 17,817 bp. The plastome contains 113 genes, consisting of 79 unique protein-coding genes, 30 unique tRNA genes, and 4 unique rRNA genes. The overall A/T content in the plastome of *C. nudiflora* is 61.90%, which the corresponding value of the LSC, SSC, and IR region were 63.80, 67.70, and 56.80, respectively.

We used RAxML (Stamatakis 2006) with 1000 bootstraps under the GTRGAMMAI substitution model to reconstruct a maximum likelihood (ML) phylogeny of 8 published complete plastomes of Rubiaceae, using *Ligustrum lucidum* and *syringa pinnatifolia* (Oleaceae) as outgroups. The phylogenetic analysis indicated that *C. nudiflora* is closer to *Lancea hirstua*. (Figure 1). Most nodes in the plastome ML trees were strongly supported. The complete plastome sequence of *C. nudiflora* will provide a useful resource for the conservation genetics of this species as well as for the phylogenetic studies for Verbenaceae.

**Figure 1.**
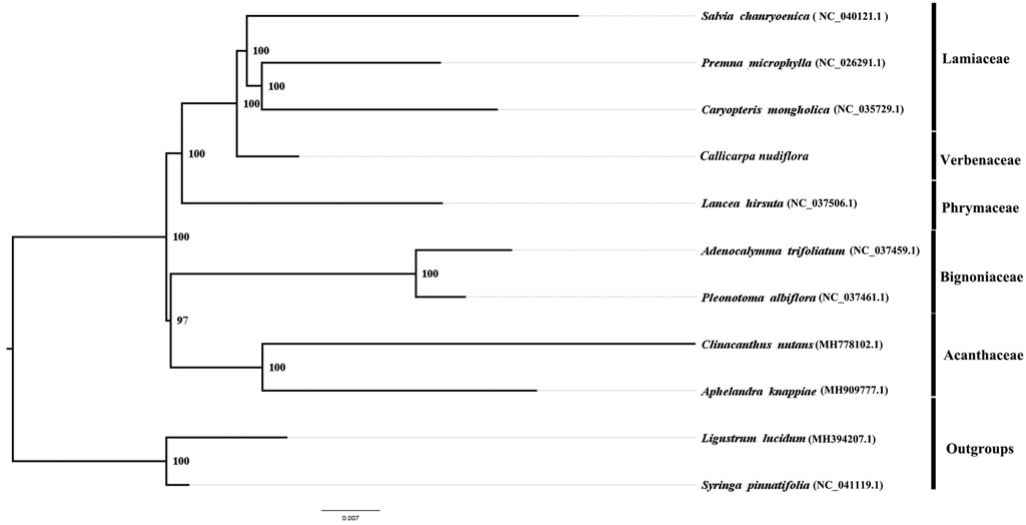
ML phylogenetic tree of *C. nudiflora*. with 8 species was constructed by chloroplast plastome sequences. Numbers on the nodes are bootstrap values from 1000 replicates. *Ligustrum lucidum* and *syringa pinnatifolia* was selected as outgroups.
